# Effects of Father-Neonate Skin-to-Skin Contact on Attachment: A Randomized Controlled Trial

**DOI:** 10.1155/2017/8612024

**Published:** 2017-01-17

**Authors:** Er-Mei Chen, Meei-Ling Gau, Chieh-Yu Liu, Tzu-Ying Lee

**Affiliations:** ^1^Keelung Hospital, Ministry of Health and Welfare, Keelung, Taiwan; ^2^Graduate Institute of Nurse-Midwifery, National Taipei University of Nursing and Health Sciences, Taipei, Taiwan; ^3^Department of Nursing, National Taipei University of Nursing and Health Sciences, Taipei, Taiwan

## Abstract

This study examines how skin-to-skin contact between father and newborn affects the attachment relationship. A randomized controlled trial was conducted at a regional teaching hospital and a maternity clinic in northern Taiwan. The study recruited 83 first-time fathers aged 20 years or older. By block randomization, participants were allocated to an experimental (*n* = 41) or a control (*n* = 42) group. With the exception of skin-to-skin contact (SSC), participants from each group received the same standard care. Both groups also received an* Early Childcare for Fathers* nursing pamphlet. During the first three days postpartum, the intervention group members were provided a daily SSC intervention with their respective infants. Each intervention session lasted at least 15 minutes in length. The outcome measure was the Father-Child Attachment Scale (FCAS). After adjusting for demographic data, the changes to the mean FCAS were found to be significantly higher in the intervention group than in the control group. We recommend that nurses and midwives use instructional leaflets and demonstrations during postpartum hospitalization, encouraging new fathers to take an active role in caring for their newborn in order to enhance father-neonate interactions and establish parental confidence. This trial is registered with clinical trial registration number NCT02886767.

## 1. Introduction

Perinatal care typically focuses on the postpartum mother and her newborn infants. Reflecting this phenomenon, most perinatal care research has focused on the mother during the pregnancy and birthing experience, excluding the feelings and functions of the father [1]. A qualitative study by Hsieh (2001) [2] found that new parents begin to experience parenting anxiety from the moment their child is born. They accumulate the experience necessary to realize their ideal parenting roles using adjustments, tradeoffs, trial and error, and external assistance. Apart from confronting the needs of new mothers and newborn infants, postnatal care delivered by nurses and midwives should benefit new fathers as well. For some men, the significance of being a father begins at childbirth [3].

Numerous studies have demonstrated the efficacy of “kangaroo care” (skin-to-skin contact, SSC) in stabilizing the blood-oxygen level, body temperature, and breathing rate of neonates. Moreover, SSC reduces infant crying, enhances infant growth and development, stimulates early breastfeeding, increases lactation, and enhances the parent-child attachment relationship [4–7]. Further, parental SSC with one's child that is performed with the intention of deepening attachment and emotional relationships has been shown to raise parental confidence toward child care [8]. Activities, such as allowing new fathers to see the face of their newborn, hug or touch their newborn, and engage in SSC, facilitate the role transition of expectant fathers [9, 10]. The first instance of intimate contact between a father and his child creates self-awareness for the former—who is a key provider for the newborn—and may further catalyze feelings of affinity and protectiveness [11].

New fathers have been shown not only to develop close emotional ties with their child 3 days postpartum, but also to invest and sustain a strong interest in him or her during this period [12, 13]. Consequently, skin-to-skin contact may help decrease parental anxiety and enhance the dependency relationship. More frequent interaction with his infant may indicate that a father is providing increased levels of positive parenting behavior as measured by the five facets: sensory stimulation, physical care, warmth, nurturing, and “fathering” [9, 10, 13]. According to Mau and Huang (2010) [14], the father plays a pivotal role in terms of family functionality, childhood development, and child well-being [14]. Children with positive father-child relationships may develop models of caregivers as trustworthy and supportive and later approach others with positive attitudes and expectations [15].

Postpartum father-neonate SSC engenders strong feelings in the father for his newborn, increases the infant's environmental stimulation, provides critical emotional support, and encourages the father to become actively involved in infant caring responsibilities [9, 10]. Touching, massaging, and hugging an infant as well as learning to respond appropriately to an infant's crying each help new fathers properly interpret changes to infant appearance and behavior; provide appropriate information; reduce parenthood discomfort and anxiety; better prepare for parenting role responsibilities; and increase infant care confidence [14, 16]. Several studies affirm that early father-neonate contact not only fosters a close father-neonate relationship, but also hastens the development of paternal attachments. These benefits suggest that fathers may assume a greater role in early postpartum parental touch when new mothers are physically weak [17, 18].

Today, it is common for expectant fathers to actively participate in the childbirth process as well as to reminisce on the experience with their spouse or others. This study implemented a SSC intervention for fathers and their newborn infants during postpartum hospitalization, observing the intervention effects on father-child attachment. Results are intended as a reference to help maternity ward staff and administrators provide high-quality, family-centered care.

## 2. Methods

### 2.1. Design

A randomized clinical trial (RCT) method was used, with research implemented under two phases. The first-phase pilot study selected 6 eligible participants who were randomly assigned to either the intervention group (*n* = 3) or control group (*n* = 3). Results of this pilot test were used to assess the appropriateness of the study questionnaire and to identify and avoid potential problems during the study's second phase, when formal survey and analysis work was conducted.

### 2.2. Setting and Samples

This study was conducted at a regional teaching hospital and a maternity clinic in northern Taiwan. Although neither site was currently Baby Friendly Hospital- (BFHI-) accredited, both were in the process of applying for BFHI accreditation at the time of this study. All of the babies were bathed at 24 hours postpartum. The traditional Chinese* doing the month *culture encourages postnatal women to stay indoors and to rest completely for one full month after birth in order to promote postpartum recovery and future health [19]. Therefore, during hospitalization, most of the babies were cared for by nurses in the nursery rather than by their mothers [20].

Participants included new fathers of vaginal and cesarean birth infants. To be eligible for inclusion, participant fathers must (1) be older than 20 years; (2) be at the hospital daily until discharge; (3) be a nonsmoker; (4) not have an alcohol addiction or be diagnosed with a psychological disorder; and (5) sign an informed consent agreement. Infant inclusion criteria included (1) gestational age** ≥** 37 weeks; (2) stable vital signs; and (3) no congenital abnormalities or diseases.

G-Power 3.1.6 software evaluated the statistical power of the study sample [21]. Based on Feldman et al. (2003) [22], the power and alpha values for the independent-samples *t*-tests were set to .8 and .05, respectively [22]. Sensitivity scores for father-child SSC for fathers averaged 4.19 (SD = .58) in the intervention group and 3.76 (SD = .78) in the control group. This generated a minimum sample of 84 participants—42 members per group. Estimating an attrition rate of 10%, this study set its target sample size at 92 participants: 46 members per group.

### 2.3. Intervention

Four field experts of clinical obstetrics and pediatrics validated the developed father-neonate SSC intervention. A description of the intervention used in this study is as follows.

The researchers facilitated initial SSC between intervention group participants and their infants within 24 hours of birthing under conditions that did not adversely affect spontaneous mother-infant SSC nor interfere with the early initiation of breastfeeding. Because it is standard practice to discharge vaginal-birth mothers on the third postpartum day, this study implemented the intervention during the first three postpartum days for both vaginal and cesarean birth cases.

Meanwhile, participants in both the intervention and comparison groups were provided with the nursing pamphlet,* Early Childcare for Fathers*, and briefed on its contents at hospital admission. Upon delivery, each newborn infant received immediate SSC with the mother and was then provided neonatal nursing before being placed temporarily into an infant crib for observation.

During the first postpartum day, after the infant was confirmed as being in a “quiet alert” state, defined as eyes open and bright, breathing normally, and being sensitive and responsive to stimuli, a researcher led the father into the nursery and helped him hold his infant. The experimental group participants were assisted to perform initial SSC, while the comparison group participants were helped only to hold their infants unless they specifically asked to be assisted to perform SSC. Immediately afterward, the researcher, in accordance with each participant's expressed preference, either withdrew from the room or observed the infant from an appropriate distance.

Two further father-infant SSC sessions were held on day 2 and day 3, respectively, in either the nursery or maternity ward for the experimental group. The sessions took place in a secluded section of the nursery or ward about two hours after one of the daily feedings and only after the infant had been bathed, towel dried, and fitted with a diaper. Bathing neonates prior to these sessions was suggested by the literature as an effective approach to put neonates into a quiet alert state, in which they are quiet but responsive to sights and sounds in their surrounding environment [23]. The session space included a comfortable armchair, a footrest, a partition screen, a pillow, and a towel or blanket. The ambient temperature was held at a constant 25~27°C.

Prior to touching their infant, participants wore a loose-fitting, front-button shirt or hospital smock and washed their hands. They then sat in the provided armchair and exposed their chest. A pillow and footrest were also made available for use. After the researcher confirmed the safety of all preparations, participants were given their infant to hold. The infant was cradled on the participant's chest in a fetal position, with the head held upwards either vertically or at a 30~60° angle. The exposed back of the infant was then covered by a blanket or clothing. The participant supported the infant with his hands placed on the infant's shoulder and back. Next, he made eye contact with the infant. Touch and soft voice contact commenced only after the infant was appropriately relaxed, as indicated by relaxed eyebrows, forehead, and chin muscles; slightly curled hands; and a comfortably curled body position [24]. Previous studies found that infants feel most at ease within 15 minutes of SSC with their parents and that verbal and nonverbal communication typically commences within this time period as well [18, 25]. Thus, the researchers defined the minimum duration of SSC sessions as 15 minutes, with sessions longer than this duration allowed to continue until either consciously ended by the father or interrupted by other infant care priorities.

The participants in the comparison group were asked to come to the nursery to learn related bathing techniques on day 2 and day 3. After bathing, these participants were helped to hold their infants. No SSC was facilitated unless a participant proactively made a request.

### 2.4. Instruments

#### 2.4.1. Demographic Information

Participant demographic variables addressed in this study included age, level of formal education, occupation, family (monthly) income, prior childcare experience, child gender preferences, participation in prenatal education, and time spent with infant during hospitalization. Infant variables addressed included gestational age at birth, parity, gender, weight, Apgar score, use of analgesics during labor, delivery type, duration of mother-infant SSC on the delivery bed, and feeding method during the first three postpartum days.

#### 2.4.2. The Early Childcare for Fathers Nursing Pamphlet

Based on a review of relevant literature and in accordance with infant developmental care principles, the researchers developed an initial draft pamphlet. Content was organized under four sections: The Nature of Newborns, Suggestions for Interacting with Your Newborn, The Benefits of Father-Infant SSC, and Care Tips and Precautions. Four field experts of clinical obstetrics and pediatrics critiqued the draft pamphlet and their suggested changes were incorporated into the final version used in this study.

#### 2.4.3. Father-Child Attachment Scale

The Father-Child Attachment Scale (FCAS) used by this study was developed by Yang (1999) [26], measuring the father's feelings or behaviors toward the baby. The self-reported scale includes 24 items with four extracted factors: exploring (10 items), touching (6 items), caring (4 items), and talking (4 items). Responses are scored on a 4-point Likert scale ranging from 1 (never) to 4 (usually). The scoring procedure summated items ranging from 24 to 96, with higher scores representing a better father-child attachment relationship. The previous studies showed an acceptable internal consistency with Cronbach's *α* values ranging .89~.95 [26, 27]. Cronbach's *α* for internal consistency was .89, with subscales of .76, .83, .91, and 87, respectively.

### 2.5. Data Collection

After receiving institutional review board (IRB) approval for this study in November 2012, a random allocation computer program generated a random stratified allocation table that was used to direct participant recruitment [28]. Upon hospital admission, participants in both the intervention and control groups received the* Early Childcare for Fathers* nursing pamphlet in order to promote understanding of early infant care and complete the self-reported pretest FCAS instrument. Afterward, intervention group participants were orally briefed on pamphlet contents and shown how to successfully perform father-infant SSC. These participants subsequently engaged in at least one ≧15-minute SSC session with their infant on each of the first three postpartum days. On the other hand, control group participants received only standard nursing care after receiving the pamphlet. After the 3-day study period, all participants completed and submitted the demographic survey form and self-reported posttest FCAS. As both groups had received SSC information, all of the participating fathers were asked to record the total time spent in SSC on each of the initial three postpartum days.

### 2.6. Data Analysis

Statistical analysis was performed using SPSS for Windows 20.0. In light of study objectives, descriptive statistics included frequency analysis and calculations of ratio, mean, and standard deviation (SD) values. Inferential statistics included independent-samples *t*-tests, paired-samples *t*-tests, and chi-square tests, with values of *p* < .05 considered significant. Variables identified in univariate analysis as having significant differences were classified as moderators, which were then introduced into the ANCOVA model in order to explore the effect of the intervention on the father-child attachment relationships after controlling for confounding variables.

## 3. Results

**Figure 1:**
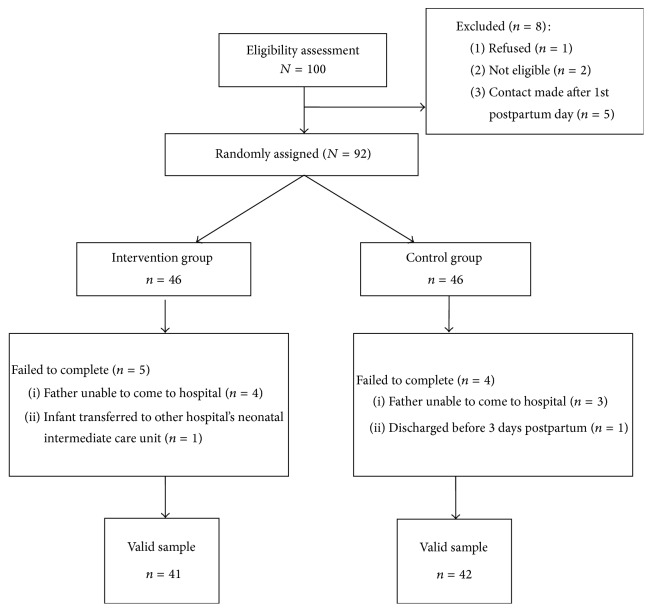
CONSORT diagram. Passage of participants through each trial stage.

A total of 100 women were assessed for eligibility; 8 were excluded due to refusal (*n* = 1) or to not meeting the inclusion criteria (*n* = 7). Ninety-two participants were randomly assigned to either the intervention group or the control group. Nine participants failed to complete the study (attrition rate: 9.8%; Figure 1). Thus, the study sample included 83 participants: 41 in the intervention group and 42 in the control group.

Table 1 provides a description of the 83 participants relative to demographic and obstetrical characteristics. The mean reported age was 34.42 years (SD = 6.70). Nearly half of participants (50.6%) were college graduates, and 56.6% were first-time fathers. They predominately lacked antenatal class attendance (85.5%), and their monthly income was greater than 75,000 New Taiwanese dollars (62.7%). About 56.6% of the participant's newborn gender was male, and 56.6% were delivered vaginally. The group demonstrated no statistical differences in demographic or obstetrical variables.

### 3.1. Comparison of Intergroup Father-Neonate Attachment Scale Results

**Figure 2:**
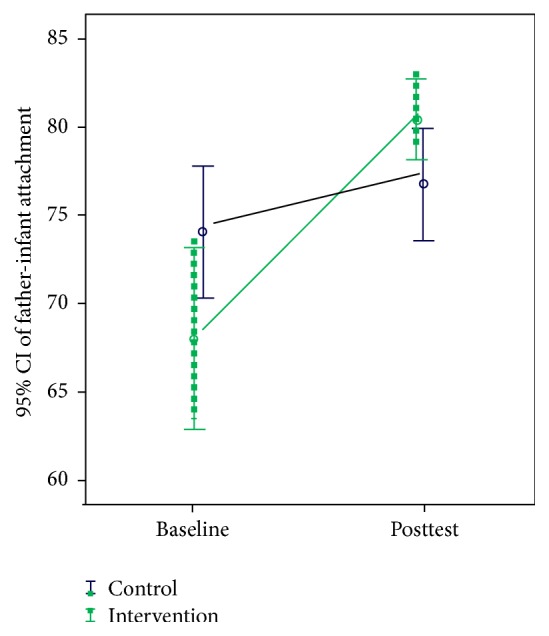
Changes in father-infant attachment scores at baseline and posttest.

Table 2 and Figure 2 show the results of statistical analyses of pretest and posttest FCAS scores for the two groups. The findings indicate that the mean posttest intervention group scores for all subscales (exploring, touching, caring, and talking) were significantly higher than corresponding pretest scores. Conversely, only the mean posttest score for touching was significantly higher than the corresponding pretest scores for the control group. Furthermore, the total pretest-to-posttest score change was larger for the intervention group than for the control group (12.44 > 2.81).

### 3.2. The Intervention Effect on the Father-Child Attachment Relationship

The demographic variables (i.e., age of the father, education level, infant gender, and time spent with the infant on days 1 to 3) that related theoretically to father-child attachment were used as the covariate variables. The covariate-adjusted effect of the intervention on the father-child attachment relationship at 3 days postpartum was derived after ANCOVA had been used to adjust for the demographic variables.

Results show that, after adjusting for covariates (Table 3), the mean posttest score for the intervention group was 6.66 points higher than that of the control group (*p* < .001). This significant difference supports the positive effect of the intervention on improving the father-child attachment relationship at 3 days postpartum.

## 4. Discussion

The study results indicate that SSC has a positive effect on paternal attachment relationship. In terms of the four FCAS subscales, mean posttest scores for subscales (exploring, touching, caring, and talking) were all significantly higher than the pretest scores for the intervention group, with the touching score displaying the largest change over time (Table 2). This finding echoes Kuan's (2012) study on the father-child attachment relationship in Taiwan, which also identified touching as the highest-scoring FCAS subscale [29].

Tarabulsy et al. (1996) identified behaviors such as hugging (physical contact), caressing, smiling, and eye contact (attentiveness/appreciation) as expressions of the attachment relationship and/or love necessary to satisfy the emotional needs of an infant and imprint feelings of intimacy and safety onto the parent-child relationship [30]. Furthermore, Goulet et al. (1998) and Young (2013) posited that physical and emotional closeness is an interactive process that reveals a commitment to love and care for a child [1, 31]. Thus, the significantly higher posttest FCAS scores earned by the intervention group in this study in comparison to the control group may reflect the positive effect of touching and hugging on these participants' understanding of their infants and their inclination to participate in childcare.

Another mechanism of the higher FCAS scores in the SSC group may in part be caused by activation of sensory stimuli and a consequent release of oxytocin [23, 32, 33]. Touch, warmth, stroking, and soft light pressure have been shown to stimulate oxytocin release and induce oxytocin-related effects [33]. Previous studies [34, 35] have found that oxytocin slows the rise in levels of stress hormones (such as catecholamines) in mothers, fathers, and newborns. Cong et al. [36] examined the role of the oxytocin mechanism in modulating parental stress and anxiety during maternal SSC (M-SSC) and paternal SSC (P-SSC). Their results showed that both maternal and paternal oxytocin levels were significantly elevated during SSC as compared with baseline and that both maternal and paternal cortisol levels were significantly elevated during SSC as compared with baseline. The authors [36] concluded that M-SSC and P-SSC activated oxytocin release and reduced stress and anxiety responses in mothers and fathers of preterm infants. Oxytocin has been shown to provoke feelings of satisfaction, to promote feelings of security and calmness, and to reduce anxiety. Furthermore, increased levels of oxytocin in the postpartum period have been shown to enhance parental-infant attachment and bonding [35, 37]. Additionally, previous studies indicate that SSC increases the self-perceived commitment of parents to their infant, with participants describing SSC as a heartwarming experience and professing a sense of fascination with the competence of their infant [31, 37]. In our study, paternal SSC also increased other relationship-facilitative behaviors, such as eye-to-eye contact, soft verbal communication, and stroking. Furthermore, all intervention group participants reported to have enjoyed the intimate-contact experience with their neonate. These results may support oxytocin as an important component of the neuroendocrinology of fatherhood [37]. However, the present study did not measure oxytocin levels. Future research may better consider and account for the impact of the intervention on oxytocin level.

The* Early Childcare for Fathers* nursing pamphlet that was provided to participants in both the experimental and control groups included the following sections: The Nature of Newborns, Suggestions for Interacting with Your Newborn, The Benefits of Father-Infant SSC, and Care Tips and Precautions. However, none of the fathers in the control group practised SSC with their neonate. Further, the control group earned significantly lower FCAS scores and spent less time with their neonates during hospitalization. This finding supports previous research showing that the stimulus-response reaction may be an important precursor to the induction and evolution of the attachment process [22]. The lack of SSC behavior in the control group may relate to “being afraid of hurting the infant” [9]. Fear of holding their infants has previously been found among parents of healthy preterm infants [38]. This situation further supports that demonstrating SSC skills is a significantly more efficacious approach to promoting SSC than providing related information via a pamphlet.

## 5. Conclusion

These study results confirm the positive effects of SSC interventions on the infant care behavior of fathers in terms of exploring, talking, touching, and caring and on the enhancing of the father-neonate attachment relationship at 3 days postpartum. Although facilitating mother-child SSC and breastfeeding immediately postpartum is standard obstetric practice in Taiwan, few fathers currently benefit from facilitated SSC with their newborn infant during postpartum hospitalization.

## 6. Clinical Implications

There are two major implications for healthcare educators, researchers, and clinicians. First, SSC interventions are a family-centered approach to healthcare that not only benefit the physiological health of infants but also facilitate the parent-child attachment relationship. Current mother/baby friendly healthcare policies stress the importance of early SSC to the well-being of mothers and their full-term infants. Expanding the skin-to-skin care approach to fathers may be an effective healthcare strategy that helps new fathers develop self-confidence and adopt a more active and positive outlook on their transition into fatherhood.

Second, the high rate of cesarean births reported in this study (56.6%) resulted in limited early skin-to-skin contact between mother and newborn directly after cesarean birth (68.7%) for practical and medical reasons. In a previous study examining SSC with the father for full-term, healthy infants, the paternal substitute was found to be as good as incubator care, according to infant body temperature after cesarean birthing [39]. As a result of these positive findings on SSC after birth, in conjunction with an increase of cesarean birth as a method of delivery and parents' equal concern for child care, postpartum care that provides for infants to be placed skin-to-skin with the father is recommended when SSC with the mother is unavailable.

Manpower limitations have prevented our researchers from approaching expectant fathers in the antenatal clinic to introduce father-neonate SSC procedures, precautions, and benefits. Instead, we were limited in providing personalized instruction to each participant immediately postpartum. A small percentage of fathers in this study expressed anxiety about the SSC intervention techniques because of a lack of preparation. Thus, we suggest that future intervention instructions be delivered in the antenatal clinic and be incorporated into formal childbirth education courses for expectant fathers in order to allow sufficient time for learning and practice.

## Figures and Tables

**Table 1:** Comparative demographic variables, intervention, and control groups.

Variable	Overall (*N* = 83) M ± SD/*n* (%)	Intervention (*n* = 41) M ± SD/*n* (%)	Control (*n* = 42) M ± SD/*n* (%)	*χ* ^2^/*t*	*p*
Age	34.42 ± 6.70	33.34 ± 5.29	35.48 ± 7.75	−1.47^a^	.146
Level of education				2.04^b^	.153
≤High school	41 (49.4)	17 (41.5)	24 (57.1)		
≥College	42 (50.6)	24 (58.5)	18 (42.9)		
Occupation				0.98^b^	.322
Unskilled	41 (49.4)	18 (43.9)	23 (54.8)		
Skilled	42 (50.6)	23 (56.1)	19 (45.2)		
Family monthly income (NT$)				0.10^b^	.755
≤75000	52 (62.7)	25 (61.0)	27 (64.3)		
>75000	31 (37.3)	16 (39.0)	15 (35.7)		
Participation in prenatal education				0.002^b^	.964
Yes	12 (14.5)	6 (14.6)	6 (14.3)		
No	71 (85.5)	35 (85.4)	36 (85.7)		
Time spent with infant during hospitalization (hours)					
Postpartum day 1	9.73 ± 8.79	11.48 ± 8.71	8.02 ± 8.64	1.82^a^	.073
Postpartum day 2	10.46 ± 8.62	11.64 ± 8.00	9.30 ± 9.13	1.24^a^	.217
Postpartum day 3	8.20 ± 6.75	9.31 ± 6.19	7.13 ± 7.16	1.49^a^	.141
Infant feeding method				2.65^b^	.266
Breast milk only	32 (38.6)	19 (46.3)	13 (31.0)		
Breast milk + formula	27 (32.5)	13 (31.7)	14 (33.3)		
Formula only	24 (28.9)	9 (22.0)	15 (35.7)		
Parity				1.52^b^	.218
Nullipara	47 (56.6)	26 (63.4)	21 (50.0)		
Multipara	36 (43.4)	15 (36.6)	21 (50.0)		
Baby gender				0.01^b^	.923
Male	47 (56.6)	23 (56.1)	24 (57.1)		
Female	36 (43.4)	18 (43.9)	18 (42.9)		
Weight (grams)	3075.75 ± 399.88	3114.73 ± 413.23	3037.69 ± 387.58	0.88^a^	.383
Delivery type				0.12^b^	.729
Vaginal	36 (43.4)	17 (41.5)	19 (45.2)		
Cesarean	47 (56.6)	24 (58.5)	23 (54.8)		
Initial mother-neonate SSC				3.31	.069
Yes	57 (68.7)	32 (78.0)	25 (59.5)		
No	26 (31.3)	9 (22.0)	17 (40.5)		
SSC during hospital stay (minutes)					
First day	—	23.85 ± 12.93	0	—	
Second day	—	24.02 ± 12.51	0	—	
Third day	—	21.80 ± 7.93	0	—	

Note: ^a^*t*-test; ^b^Pearson chi-square; ^c^Linear-by-linear association; SSC: skin-to-skin contact.

**Table 2:** Pretest and posttest FCAS scores and score changes (*N* = 83).

Variable	Intervention	Control	*t* ^2^ (*p*)
	M ± SD	M ± SD	
*Exploring*			
Pretest	3.40 ± 0.59	3.57 ± 0.39	1.45 (.152)
Posttest	3.69 ± 0.32	3.66 ± 0.38	−0.50 (.616)
*t*^1^/(*p*)	3.89 (<.001)	1.83 (.074)	
*Talking*			
Pretest	2.95 ± 0.74	3.11 ± 0.66	1.03 (.305)
Posttest	3.42 ± 0.48	3.30 ± 0.64	−0.93 (.357)
*t*^1^/(*p*)	4.67 (<.001)	2.12 (.033)	
*Touching*			
Pretest	2.63 ± 1.03	3.02 ± 0.79	1.94 (.056)
Posttest	3.50 ± 0.47	3.21 ± 0.67	−2.31 (.024)
*t*^1^/(*p*)	6.20 (<.001)	2.36 (.023)	
*Caring*			
Pretest	2.92 ± 0.81	3.23 ± 0.60	1.98 (.051)
Posttest	3.41 ± 0.50	3.27 ± 0.44	−1.34 (.200)
*t*^1^/(*p*)	4.85 (<.001)	0.54 (.591)	
*FCAS*			
Pretest	68.00 ± 16.39	74.17 ± 11.99	1.94 (.056)
Posttest	80.44 ± 7.26	76.98 ± 10.19	−1.76 (.081)
*t*^1^/(*p*)	5.92 (<.001)	2.45 (.020)	

Notes: FCAS: Father-Child Attachment Score; *t*^1^ compares intragroup differences (pre/posttest) using paired-samples *t*-tests.

*t* ^2^ compares intergroup differences (intervention/control) using independent-samples *t*-tests.

**Table 3:** Comparative effect of father-child skin-to-skin contact on the father-child attachment relationship.

Variable	*B*	SE	95% CI	*t*	*p*
Intercept	53.57	3.69	46.23~60.91	14.53	<.001
Baseline	0.39	0.05	0.29~0.50	7.49	<.001
Group^*∗*^					
Intervention	6.66	0.05	3.56~9.76	4.28	<.001
Control	Reference				

^*∗*^Adjusted for age and education level of father, gender of infant, and time spent with the infant on days 1 to 3.
